# Supplementary material for: Bridging the gap between single-cell migration and collective dynamics
Source: eLife. 2019 Dec 6;8:e46842. doi: 10.7554/eLife.46842 (PMC6992385; doi:10.7554/eLife.46842)
Supplement: Source code 1. [file elife-46842-code1.tar › Source Files/README.rtf]

Software requirements: HDF5, Boost, GSL, GCCCOMPILATION:All Source Code files are compiled using CMake, by running the following commands in the respective folders (in “Simulation Code” and “Processing Code“):cmake CMakeLists.txtmakeCONFIGURATION FILES:All configuration files are provided in “Simulation Parameters”. Figures are associated with their corresponding configuration files in “Figure_List.xlsx”. To rerun a simulation, go to the corresponding simulation folder (e.g. “Simulation Parameters/single_Q/pars_0/rng_1/”). Place the corresponding Substrate Pattern File (see Figure_List.xlsx) in the simulation folder and rename it to “CustomPattern.h5”. Then, place the compiled binary from “Simulation Code“ in the simulation folder and run it. Preprocessing of the Simulation Code is done with the corresponding binary from “Processing Code”. Processing of the Simulation Code is done with the corresponding Mathematica Notebook.CAUTION:When computing correlation functions, cell velocities lower than 10e-5 should be rounded to zero or discarded (lower cell speed are not possible due to the discretization). This can become an issue when computing normalized correlation functions.The parameter CONFINEMENT_RADIUS actually denotes the squared confinement radius.In the ‘rotation’ datasets, one can only measure an angular velocity if the cell count is larger than or equal to 2.
